# Eleven fetal echocardiographic planes using 4-dimensional ultrasound with spatio-temporal image correlation (STIC): a logical approach to fetal heart volume analysis

**DOI:** 10.1186/1476-7120-8-41

**Published:** 2010-09-15

**Authors:** Surasak Jantarasaengaram, Kittipong Vairojanavong

**Affiliations:** 1 Maternal-Fetal Medicine Unit and Ultrasound Unit, Rajavithi Hospital, College of Medicine, Rangsit University, Bangkok, Thailand

## Abstract

**Background:**

Theoretically, a cross-sectional image of any cardiac planes can be obtained from a STIC fetal heart volume dataset. We described a method to display 11 fetal echocardiographic planes from STIC volumes.

**Methods:**

Fetal heart volume datasets were acquired by transverse acquisition from 200 normal fetuses at 15 to 40 weeks of gestation. Analysis of the volume datasets using the described technique to display 11 echocardiographic planes in the multiplanar display mode were performed offline.

**Results:**

Volume datasets from 18 fetuses were excluded due to poor image resolution. The mean visualization rates for all echocardiographic planes at 15-17, 18-22, 23-27, 28-32 and 33-40 weeks of gestation fetuses were 85.6% (range 45.2-96.8%, N = 31), 92.9% (range 64.0-100%, N = 64), 93.4% (range 51.4-100%, N = 37), 88.7%(range 54.5-100%, N = 33) and 81.8% (range 23.5-100%, N = 17) respectively.

**Conclusions:**

Overall, the applied technique can favorably display the pertinent echocardiographic planes. Description of the presented method provides a logical approach to explore the fetal heart volumes.

## Background

Fetal heart examination is based upon visualization of certain cross-sectional planes or views on conventional two-dimensional (2D) ultrasound scanning. The abdominal view, the four-chamber view, and both cardiac outflow tracts should be obtained at screening level[1]. Additional fetal cardiac views are crucial for the sequential segmental analysis to assess the connections and anatomical detail[2-6]. Unfortunately, the fetal heart is not only small but also beating at high rates. Fetal heart geometry is quite complex, so it needs the three-dimensional (3D) mental reconstructions to understand chambers-vessel spatial connections and relationships. Furthermore, directing the ultrasound beam in the correct plane is complicated by different fetal positions. Therefore, a specific training and a considerable scanning experience are required for an examiner to be able to display the pertinent fetal cardiac planes.

Progress in diagnostic ultrasound using the volume ultrasound and spatio-temporal image correlation (STIC) technique enables examiners to generate a volume dataset of the fetal heart with cardiac motion. Along with the multiplanar display mode, a cross-sectional dynamic 2D image in any plane can also be obtained. Theoretically, with a good quality volume dataset, any examiner can navigate inside the volume to display the pertinent diagnostic cardiac planes, irrespective of an operator's scanning skill.

A concept of initial adjustment of the volume dataset to a uniform virtual organ or fetal orientation (standardization) prior to exploring into the volume has been suggested to ease three-dimensional spatial orientation and enable a reproducible diagnostic plane and image from the corresponding movement and volume analysis technique[7,8]. Recently, a computer software that can automatically display cardiac outflow tracts after standardization of the fetal chest volume has been successfully validated[9,10]. We present a method to adjust virtual fetal position in STIC fetal heart volume datasets to a uniform fetal orientation (standardization of virtual fetal position) and a systematic approach to navigate in the standardized volumes to display the following 11 echocardiographic planes: (1) abdominal view; (2) four-chamber view; (3) five-chamber view; (4) three-vessel view; (5) three-vessel and trachea view; (6) transverse aortic arch; (7) long axis of the ductal arch; (8) long axis of the aortic arch; (9) long caval view; (10) left ventricular outflow tract (LVOT); and (11) short axis view of the great vessels. The concept of the presented method simplifies spatial perception of cardiac anatomy and provides a logical way to analyze the STIC fetal heart volumes in the multiplanar display modality.

## Methods

Pregnant women from 15 weeks of gestation who attended our antenatal care clinic and needed an obstetric ultrasound scanning of any indication were invited to add to their scanning a volume ultrasound examination of the fetal heart using the STIC technique. The local Ethic Committee has approved the study prior to enrolling the women. After signing a written consent, the participating women were examined by our routine obstetric ultrasound protocol. Fetuses with abnormal four-chamber view and/or abnormal cardiac outflow tracts were excluded. The study included 200 fetuses from 15 to 40 weeks of gestation. The primary author acquired STIC volume datasets with transverse sweeps through the fetal chest at appropriate scanning window for viewing the heart. The 2D image was adjusted for the highest resolution by using as high as probe's frequency as possible. In order to obtain high frame rate, the scan area was set to cover only the fetal thorax in transverse plane and single focal point was used. The dynamic range was adjusted for high contrast image. Adjustments to the region of interest (ROI) box provided coverage of the fetal spine and sternum in the fetal axial plane. The time for acquiring the volume datasets was limited within 15 minutes in each case.

Volume acquisition was performed with Voluson 730 Expert or Voluson 730 ProV (GE Healthcare, Milwaukee, WI, USA) with transabdominal mechanical probe RAB 5-7 L. Depending on the fetal movements and its gestational age, the acquisition time ranged from 7.5 to 15 seconds, and the angle of acquisition ranged between 15 to 40 degrees. The four-chamber view or any transverse planes adjacent to the four-chamber view served as a starting point for acquisition. Three to six volumes were generated from each fetus. The volume datasets were stored in an external hard disk and analyzed offline on a computer several days later by the primary author using the software 4D View Version 5.0 (Kretztechnik, Zipf, Austria). In each fetus, volume datasets were consecutively retrieved for analysis in the multiplanar display mode based on their image quality in plane B. The total number of volume datasets used to display as many cardiac planes as possible in each fetus was recorded. Visualization rates of each cardiac view are determined. In case that the volumes analysis failed to display a normal four-chamber view, right or left outflow tracts, the woman was contacted to come back for a standard fetal echocardiographic examination.

**Figure 1:**
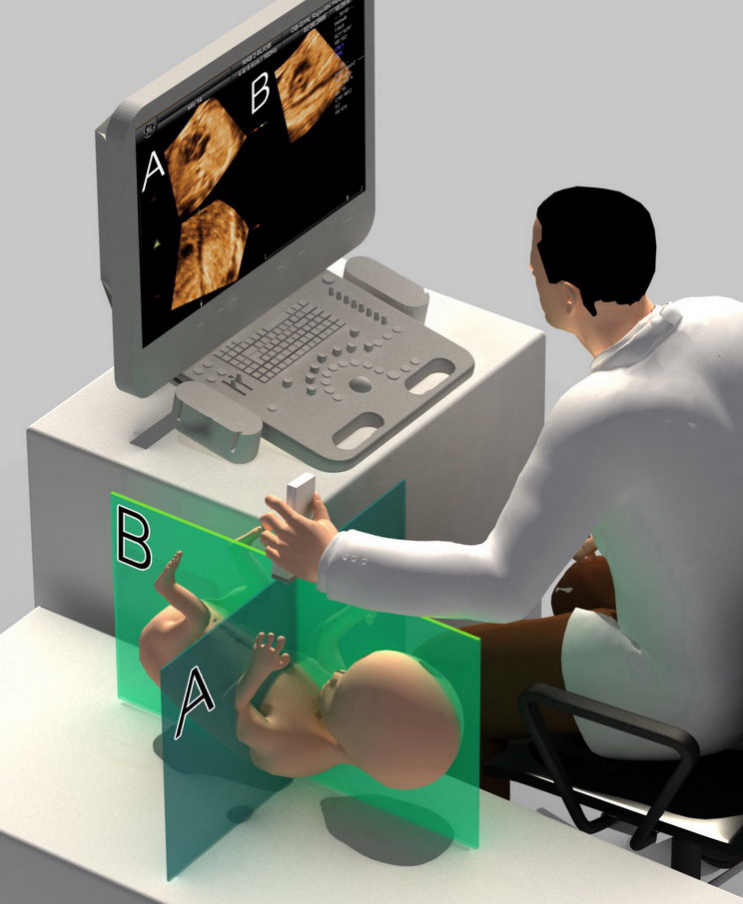
**After standardization of fetal position in the STIC volume dataset to place the fetus in the exact dorsal supine position, navigating in the volume is similar to moving the transducer over the fetal chest in real time 2-dimensional scanning**. The transverse planes are displayed in plane A and the saggital planes are displayed in plane B.

**Figure 2:**
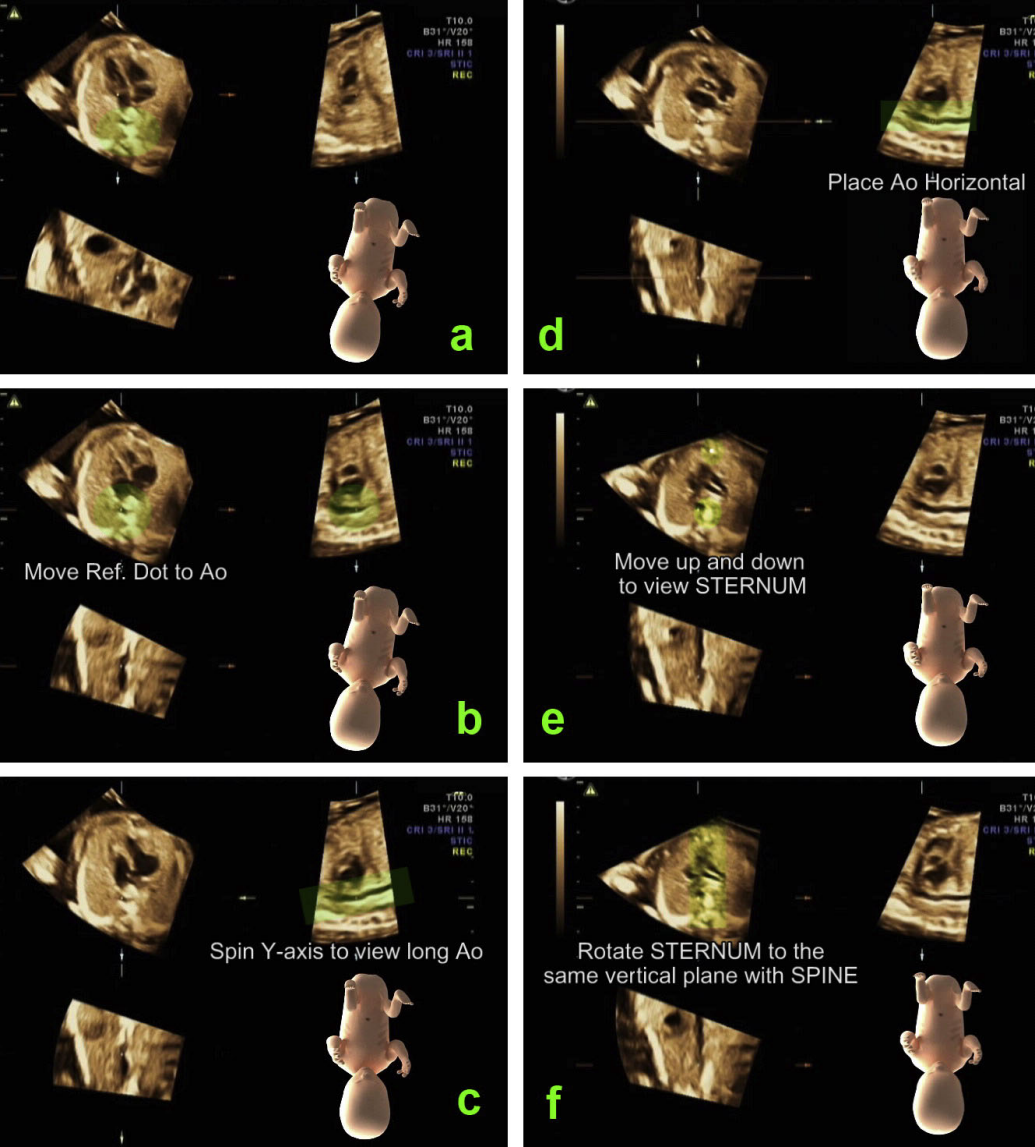
**This image illustrates the steps to standardize virtual fetal position in order to place the fetus in the "exact dorsal supine position"**. a. In plane A, rotate the spine down to six o'clock. b. In plane A, move the reference dot to the center of the aorta. c. In plane B, spin the volume around the y-axis to display long axis of the descending aorta d. In plane B, rotate the volume around the z-axis to place the descending aorta horizontally. (According to this movement, the volume is placed as if the fetus was lying down on a flat table. However, the fetus may not lie in the exact supine position but more or less on its side.) e. Move to plane A and scrolling parallel up and down through the volume until the sternum echo is depicted. f. Rotate the image in plane A around the z-axis until the sternum and the spine are in a vertical alignment. This last movement results in the exact dorsal supine fetal position.

The principle underlying the presented technique is to initial manipulate the volume dataset to place the fetus in a standardized position as if it is lying horizontally on an examination bed in the "exact dorsal supine position". Then, navigating in every standardized volume in the multiplanar display mode is similar to moving the transducer over the chest of the lying fetus. The examiner can visualize the cardiac planes that are necessary to scan the fetal heart in the transverse plane by scrolling in plane A and in the longitudinal plane by scrolling in plane B (Figure 1). This concept offers a sonographer a more comprehensible orientation while navigating in the volume dataset. The details of this technique are described in the Appendix, Figure 2 and Additional file 1 for the fetus in a cephalic presentation and the Additional file 2 for the fetus in a breech presentation.

## Results

The volume datasets from 18 fetuses were excluded due to poor image resolution. Of the remaining 182 fetuses, the mean gestational age was 23.6 ± 6.1 weeks. The most common indication for ultrasound examination was routine obstetric screening (50.5%). A total of 420 volume datasets were used for the systematic approach (mean number of volumes = 1.73 ± 0.66, range 1-3 per one fetus). The four-chamber view was not clearly displayed in one fetus at 15-17 weeks of gestation and in another fetus at 18-22 weeks of gestation. The three-vessel view was not clearly displayed in five fetuses at 15-17 weeks of gestation and in one fetus at 18-22 weeks of gestation. Furthermore, the LVOT could not clearly displayed in four fetuses at 15-17 weeks of gestation and in one fetus at 18-22 weeks of gestation. All these fetuses were rescanned one to three weeks later, and all of them had a normal fetal echocardiography result. Table 1 shows the visualization rates of the 11 echocardiographic planes at various gestational age groups. Visualization rates of the abdominal view and the four-chamber view in all gestational age groups were not significantly different. Visualization rates of the other echocardiographic planes were significantly different among gestational age groups. The mean visualization rates for all echocardiographic planes in the 33-40 week's gestation fetuses and 15-17 week's gestation fetuses are relatively low.

**Table 1:** Visualization rates of fetal echocardiographic planes at various gestational ages

Echocardiographic planes		15-17 wk (N = 31)	18-22 wk (N = 64)	23-27 wk (N = 37)	28-32 wk (N = 33)	33-40 wk (N = 17)	Total N = 182	*p *value
Abdominal view	%	96.8(30/31)	100(64/64)	97.3(36/37)	97.0(32/33)	100(17/17)	98.2	1.000
4CH view	%	96.8(30/31)	98.4(63/64)	100(37/37)	100(33/33)	100(17/17)	99.0	0.606
5CH view	%	87.1(27/31)	100(64/64)	100(37/37)	100(33/33)	100(17/17)	97.4	0.002
3VV view	%	83.9(26/31)	98.4(63/64)	100(37/37)	100(33/33)	100(17/17)	96.5	0.003
3VT view	%	45.2(14/31)	64.1(41/64)	81.1(30/37)	69.7(23/33)	82.4(14/17)	68.4	0.016*
Transv Ao arch	%	74.2(23/31)	76.6(49/64)	51.4(19/37)	54.5(18/33)	23.5(4/17)	56.0	<0.001*
Long ductal arch	%	93.5(29/31)	98.5(63/64)	100(37/37)	84.8(28/33)	64.7(11/17)	88.3	<0.001
Long Ao arch	%	96.8(30/31)	95.3(61/64)	100(37/37)	93.9(31/33)	76.5(13/17)	92.5	0.022
Long caval view	%	93.5(29/31)	96.9(62/64)	97.3(36/37)	78.8(26/33)	70.6(12/17)	87.4	0.001
LVOT	%	87.1(27/31)	98.4(63/64)	100(37/37)	100(33/33)	100(17/17	97.1	0.014
Short axis Great V	%	83.9(26/31)	95.3(61/64)	100(37/37)	97.0(32/33)	82.4(14/17)	92.3	0.016
Mean of all views	%	85.6	92.9	93.4	88.7	81.8		

4CH = four-chamber, 5CH = five-chamber, 3VV = three-vessel, 3VT = three-vessel and trachea, Transv Ao arch=transverse aortic arch, Long ductal arch=long axis of ductal arch, Long Ao arch = long axis of aortic arch, Long caval = longitudinal caval, LVOT = left ventricular outflow tract and Short axis Great V = short axis view of the great vessels.

*p *value from Fisher's Exact Test and *p *value* from Pearson Chi-square

## Discussion

Standardization of fetal position in the volume dataset is critical for volume analysis. Due to the small size of the fetal heart, minimal deviation from a certain cross-sectional plane would prohibit visualization of the required diagnostic image. Thus, we make a minor modification of the standardization of virtual fetal position method from the technique that has been described by Abuhamad[7]. The reference dot is placed over the descending aorta in plane A instead of the spine (body of vertebra) (Figure 2b) in order to obtain the precise longitudinal alignment. In addition, vertical alignment of sternum and spine is included in the process for precise transverse alignment (Figure 2f). The "exact" dorsal supine virtual fetal position enables the development of a road map that is the systematic approach to access into the pertinent cardiac planes.

Using the systematic approach after standardization of virtual fetal position, the visualization rate of each cardiac plane was favorable and could be comparable with the previously reported techniques[10-13]. In the small fetuses at 15-17 weeks of gestation, the overall visualization rates were relatively lower due to the small heart and small size of vessels. In the third trimester, fetal position rarely changes over time. Acoustic shadows from the fetal shoulder, scapula, ribs and upper extremities might obscure the image resolution in the upper mediastinum during volume acquisition. This was responsible for the lower visualization rates of transverse aortic arch, long axis of the ductal arch and long axis of the aortic arch in the large fetuses. To visualize the short axis views using the systematic approach, we navigated perpendicularly to the descending aorta from the fetal abdomen up to the mediastinum (Appendix, step 2-step 7). The normal fetal flexion altitude facilitated the display of the three-vessel and trachea view which is an angulated views in some but not all fetuses, giving the lower visualization rates of the three-vessel and trachea view when compared to the axial views (abdominal view, four-chamber view, five-chamber view and three-vessel view).

Goncalves et al. [14] has described an approach to visualize the left fetal cardiac outflow tract and short axis view of the pulmonary artery after adjustment of the A-plane image in the multiplanar display mode to a perfect four-chamber view. The same group has subsequently added topographic ultrasound imaging (TUI) display to their technique to visualize more echocardiographic planes including: the five-chamber view, the three-vessel and trachea view and longitudinal view of the ductal arch[13]. DeVore et al. [15] has introduced the "spin" technique to display the entire vessel by placing a reference point over the vessel of interest and spinning the A-plane image until the full length of each vessel is identified. The technique needs visualization of four initial axial cardiac planes (the four-chamber view, the five-chamber view, the three-vessel view and the three-vessel and trachea view) in the A-plane prior to rotation of the volumes around the X-, or Y-axis to open up the vessel of interest. Unfortunately, because of different fetal positions, an optimal scanning window while activating a transverse volume acquisition is infrequently perpendicular to the fetal thorax. Thus, the optimal four-chamber view and the relevant initial axial cardiac images are not instantaneously displayed on the A-plane panel. An examiner who is not a STIC expert nor a fetal echocardiologist will have a hard time adjusting the volume to get the proper initial A-plane images at the outset of volume manipulation.

The presented technique offers some advantages over the previously reported techniques[13-15]. It can be applied to analyze fetal heart volumes that were acquired from any transverse planes. A sonographer can start the volume acquisition from any transverse cut that provides a good heart image. The acquiring planes need not to be perpendicular to the fetal thorax and the starting point of acquisition need not to be at the four-chamber view. After adjustment of virtual fetal position in a volume dataset to the exact dorsal supine position, scrolling along plane A automatically displays the required axial planes (the abdominal view, the four-chamber view, the five-chamber view, the three-vessel view and transverse aortic arch). The ability to acquire a STIC volume from variable transverse cuts enhances volumes' image quality. In addition, using the systematic approach, the fetal heart can be sequentially examined under continuous movements along Plane A and B, which is similar to scanning the fetal heart with conventional 2D ultrasound. Scrolling from the fetal abdomen through the base of a moving heart up to the great vessels in plane A (Appendix, step 2-step 7) allows the examiner to perceive the spatial relations (situs, outflow tracts coming out from the corresponding chambers, criss-crossing of the great vessels and position and size of those vessels) under a three-dimensional mental reconstruction.

The applied technique is also helpful to display difficult 2D longitudinal scanning planes (long axis of the aortic arch and long axis of the ductal arch). Although there is a belief that these longitudinal views should be obtained from an oblique or sagittal acquisition, there has been no substantiate objective data. Nonetheless, Bega et al. [16] acquired 3D static volume from 18 fetuses between 16 and 26 weeks of gestation using four different acquisition planes. They successfully obtained the aortic arch and the ductal arch in 66 and 86 percent, respectively, from the transverse four-chamber view acquisition and in 57 and 71 percent, respectively, from the left parasagittal acquisition. Our results also showed high visualization rates of the long axis of the ductal arch and long axis of the aortic arch from the transverse volume acquisition.

**Figure 3:**
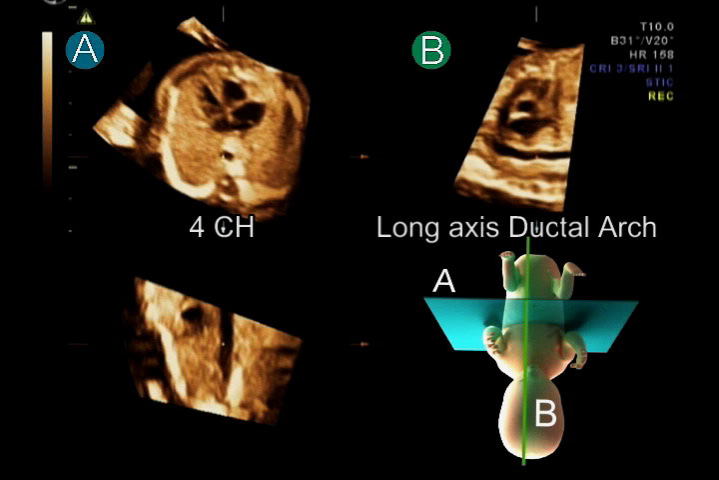
**After standardization of fetal position in the STIC volume dataset to place the fetus in the exact dorsal supine position, navigating systematically in the volume usually provides a reproducible image from a corresponded movement**. Placing the reference dot in the center of the aorta in the four-chamber (4 CH) view in plane A simultaneously displays long axis of the ductal arch in plane B.

**Figure 4:**
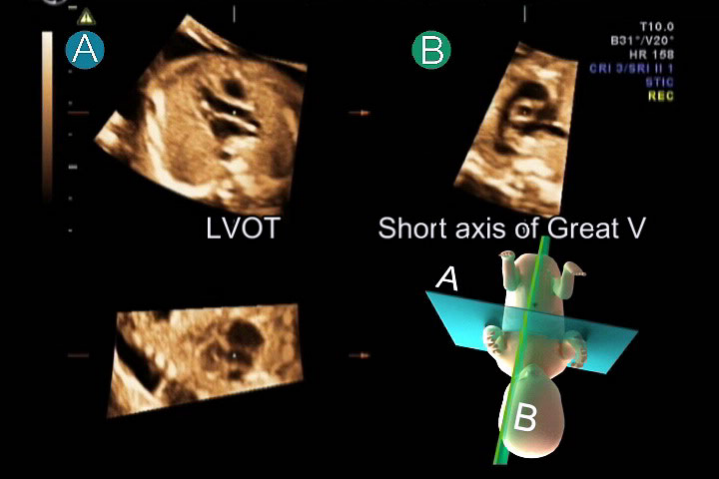
**After standardization of fetal position in the STIC volume dataset to place the fetus in the exact dorsal supine position, navigating systematically in the volume usually provides a reproducible image from a corresponded movement**. Placing the reference dot at the aortic valve in the five-chamber view in plane A and spin the volume around y-axis 15-30 degrees simultaneously displays the LVOT in plane A and short axis of the great vessels in plane B.

Clinically, STIC technique in the multiplanar display modality is rarely useful for a fetal echocardiologist who can usually guide the ultrasound beam to display the required cardiac planes irrespective of fetal positions. However, it can help a sonographer who is not familiar with fetal cardiac scanning retrieving the pertinent cardiac planes from a fetal heart volume dataset[17,18]. The presented method will shorten a learning curve in STIC volume analysis. The underlying concept simplifies spatial perception of the fetal cardiac anatomy. Once fetal position is standardized, navigating systematically in the volume dataset usually provides a reproducible image from a corresponded movement (Figures 3 and 4). According to these reasons, we also encourage this method for training and educational purpose.

### Limitations

Volume datasets were acquired and analyzed by single operator. Further study is need for validation of this technique.

## Conclusions

We described a method to standardized virtual fetal position in the STIC fetal heart volume dataset and a systematic approach to navigate in the standardized volume, which can favorably display eleven fetal echocardiographic planes. Description of the presented technique provides a logical approach to explore the fetal heart volume dataset in the multiplanar display mode.

## Competing interests

The authors declare that they have no competing interests.

## Authors' contributions

SJ carried out acquiring and analyzing the volume datasets and participated in preparing and revising the manuscript. KV participated in preparing and revising the manuscript. All authors read and approved the final manuscript.

## Appendix

Description of the technique used for standardization of fetal position and the systematic approach to display eleven echocardiographic planes.

### 1. The technique in a cephalic presentation fetus (Additional file 1)

#### 1.1 Standardization of fetal position

##### Step 1: Placing the fetus in the exact dorsal supine position

In plane A, rotate the spine down to six o'clock. Move the reference dot to the center of the aorta. In plane B, spin the volume around the y-axis to display long axis of the descending aorta. Then, rotate the volume around the z-axis to place the aorta horizontally. At this moment, the volume is placed as if the fetus was lying down on a flat table. However, the fetus may not lie in the exact supine position but more or less on its side. Placing the fetus in the exact supine position is to be accomplished by moving to plane A and scrolling parallel up and down through the volume until the sternum echo is depicted. Then, with the reference dot in the center of the aorta, rotate the image in plane A around the z-axis until the sternum and the spine are in a vertical alignment.

#### 1.2 Systematic approach to navigate in the standardized volume dataset

##### Step 2: Abdominal view (spine, stomach and inferior vena cava)

Start with moving down to the fetal abdomen by scrolling parallel in plane A across the fetal diaphragm. This movement automatically reveals the abdominal view, which displays the relationship between fetal spine, stomach and inferior vena cava (IVC).

##### Step 3: Four-chamber view

Move up by scrolling parallel in plane A across the fetal diaphragm until the four-chamber view is visible.

##### Step 4: Five-chamber view

In plane A, scroll up just above the four-chamber view level. The five-chamber view that displays the aortic root appears between the interventricular septum or the aortic root coming out of the left ventricular chamber is depicted.

##### Step 5: Three-vessel view

Scrolling parallel further up along plane A reveals the main pulmonary artery coming out of the right ventricular chamber in the three-vessel view. The ascending aorta and superior vena cava (SVC) are on the right side of the pulmonary artery.

##### Step 6: Three-vessel and trachea view

Move slightly further up by scrolling parallel along plane A. The ductus arteriosus is visualized as an extending vessel from the pulmonary artery. The aortic arch and SVC are on the right side of the duct. The duct and the aortic arch merge at the descending aorta. The trachea is seen in front of the spine.

##### Step 7: Transverse aortic arch

Scrolling further up along plane A, the transverse aortic arch is depicted.

##### Step 8: Long axis of the ductal arch

In plane A, scroll back down to the level of the four-chamber view. Long axis of the ductal arch is simultaneously displayed in plane B (Figure 3). In some cases, minimal adjustment may be required by a slight rotation of the image in plane A around the z-axis.

##### Step 9: Long axis of the aortic arch

In plane A, rotate the image in Step 8 around the z-axis to the left side of the examiner until the foramen ovale and the aorta are in a vertical plane (30-45 degree counterclockwise). Long axis of the aortic arch is simultaneously displayed in plane B.

##### Step 10: LVOT and Short axis of the great vessels

In plane A, rotate the volume back to the dorsal supine position (Step 8). Scroll up to the five-chamber view. Move the reference dot to the aortic valve and spin around the y-axis 15-20 degrees to open up the LVOT. Short axis of the great vessels is simultaneously display in Plane B (Figure 4).

##### Step 11: Long caval view

In plane A, spin around the y-axis back to the five-chamber view. Move down along plane A across the fetal diaphragm to the abdominal view that displays the stomach, spine and IVC. Move the reference dot from the aorta to the IVC. The long caval view is visualized in plane B. (Minimal y-axis adjustment in plane B may be required.)

### 2. The technique in a breech presentation fetus (Additional file 2)

Regarding the four-chamber view in plane A, the apex of the heart usually points to the left side of the examiner in a cephalic presentation fetus and to the right side in a breech presentation fetus. For a breech presentation fetus, spinning the volume in plane A around the y-axis 180 degrees yields the same image as in a cephalic presentation fetus (the apex points to the left side of the examiner). Then the steps to manipulate and navigate in the volume are the same as in a cephalic presentation fetus.

## Supplementary Material

Additional file 1 **This video clip illustrates the technique used to standardization of virtual fetal position in 4D STIC volume datasets and the systematic approach to the standardized volume to display 11 standard cardiac views in a cephalic presentation fetus**. Click here for file

Additional file 2 **This video clip illustrates the technique used to standardization of virtual fetal position in 4D STIC volume datasets and the systematic approach to the standardized volume to display 11 standard cardiac views in a breech presentation fetus**. Click here for file
